# Potential predictors of hospital length of stay and hospital charges among patients with all-terrain vehicle injuries in rural Northeast Texas

**DOI:** 10.5249/jivr.v12i1.1219

**Published:** 2020-01

**Authors:** Anastasia Miller, Jeanie D. Gallegly, Gabriela Orsak, Sharon D. Huff, Jo Ann Peters, Jason Murry, Harrison Ndetan, Karan P. Singh

**Affiliations:** ^*a*^ Department of Health Care Administration, Texas Woman's University, USA.; ^*b*^ University of Texas Health Science Center at Tyler, Tyler, Texas, USA.; ^*c*^ Department of Epidemiology and Biostatistics, University of Texas Health Science Center at Tyler, Tyler, Texas, USA.; ^*d*^ Department of Surgery, UT Health East Texas-Tyler, Tyler, Texas, USA.

**Keywords:** All-terrain vehicles, Length of stay, Traumatic injury, Hospital charges, Rural health

## Abstract

**Background:**

All-Terrain Vehicles (ATVs) have become popular for recreation use in recent years. Texas has had more ATV related fatalities than any other state in the nation, with rural Northeast Texas having even higher rates of injuries. There is limited data examining the relationship between ATV injuries and the length of hospital stay, as well as hospital costs. This paper examines both issues in children as well as adults.

**Methods:**

The regional trauma registry was analyzed for all ATV related injuries between January 2011- October 2016. Injury Severity Score, Glasgow Coma Scale and if they are seen at a Level I Trauma center are predictive for both hospital length of stay and charges.

**Results:**

Length of Stay was predicted positively by Injury Severity Score, Emergency Department Respi-ration Rate and facility at which patients were treated and negatively by Glasgow Coma Scale. Hospital charges were predicted positively by age, Injury Severity Score, facility of treatment, means of transportation, and Emergency Department pulse and negatively by Glasgow Coma Scale.

**Conclusions:**

The study found that vital signs can be useful in predicting length of stay and hospital charges. This study not only confirms the findings of other studies regarding what predictors can be used, but expands the research into rural traumatic injuries. It is hoped that this data can help contribute to the development of algorithms to predict which patients will be most likely to require resource intensive treatment.

## Introduction

Northeast Texas is a primarily rural region, approximately the size of West Virginia.^1^ Common activities often include the use of all-terrain vehicle (ATV). While initially designed for work-related use on farms, they have become popular for use in recreation and sports. ^2^ However, there have been more ATV related fatalities in Texas than any other state in the nation.^3^ In addition to this, Northeast Texas has a higher rate of ATV-related injuries in children under the age of eighteen when compared to Texas as a whole.^4^ Injuries mostly occur among adults, however, there has been a nationwide increased prevalence of accidents occurring in children under the age of 16.^3^ Potential contributing factors for all age groups include the rurality of the region and lack of restriction on helmet use,^5^ which contributes to significant head and face injuries.^6^ This is a potentially expensive avoidable injury, in both financial and human terms. 

Larger ATVs can be over 800 pounds and capable of speeds in excess of 80 miles per hour.^7^ Behaviors of ATV drivers can determine the occurrence and severity of ATV-related wrecks^8^ such as: the increased power and size of ATVs, drug and alcohol use, carrying passengers, and driving on paved or public roads.^9^ About a quarter of all U.S. related crashes involve children under the age of sixteen^7^ with children under the age of 11 years being more likely to be passenger victims.^10-11^ Rural populations exhibit disproportionately high injury mortality rates.^12^ There are many factors which contribute to the disparities in not just injury rate but in the impact of injury on rural populations.^13^ Some are behavioral aspects associated with rurality such as increased use in alcohol, decreased use of seat belts and increased access to firearms.^12^ Some aspects are factors of the geographic reality of living in a rural setting, such as the physical distance to care, longer time to discovery of injury by others, the added time of emergency services and often lack of trauma care.^14^ Another issue faced by rural populations is an access to care, including disparities in coverage of health insurance and financial barriers to care.^15^ This makes the costs of particular interest, as they are either shouldered directly by the patient, provided as uncompensated care, or some combination thereof. This is of particular public health and policy concern to those in remote and rural regions disproportionally hit with unintentional injuries^16^ and lack of health insurance, such as Northeast Texas.^1^

The vast majority of the previous articles examining ATV injuries focused exclusively on pediatric injuries. ^17-18^ In fact, the authors could only find four other articles during the last 20 years, which included adults in the analysis,^19-20^ only two of which examined hospital length of stay. ^21-22^ The authors also would like to fill a gap, and examine which factors are predictive of hospital length of stay (LOS) and hospital charges in ATV injuries in a rural setting. ATV injuries can be expensive, with estimated costs of $8,802.90 for the traumatic injuries incurred.^23^ However, there has been minimal literature examining the use of hospital resources (as approximated by length of stay) or the costs incurred by these injuries. This type of information can help shed light on the severity of this issue beyond mere indecent rates. This information would be useful to public health practitioners, policy makers, and health care administrators. Therefore, the purpose of this paper is to examine what factors associated with ATV injuries can be used to predict hospital length of stay and costs. By doing this, it is hoped that hospitals can more quickly identify which patients are likely to need greater resources and policy makers can uncover mitigating factors to target to reduce the burden of these injuries in the long term.

Commonly used measures to gauge traumatic injuries include the Injury Severity Score (ISS),^24^ the Glasgow Coma Scale (GCS),25 the shock index.^26^ The initial vital signs upon arriving in the Emergency Department (ED) are also often early indicators of severe problems and future adverse events.^27^ The ISS is currently the most widely used method of assessing severity of injury in blunt trauma. ^28^ The use of the Injury Severity Score (ISS) has been used to assess the severity of ATV injuries before in the literature.^29^ The scale ranges from 1 to 75, with scores over 16 associated with a 10% increased mortality risk.^30^ The GCS is used to assess the neurological functioning of individuals. The scale ranges from 3 to 15, with high scores indicating increased neurological responses to external stimuli.^31^ Initial emergency department vital signs have been used before to predict trauma mortality, although the usefulness of simple vitals has been called into question.^32^ Finally, indicators based on vital signs may prove more useful such as the shock index. The shock index (heart rate/systolic blood pressure) can be used to predict the severity of hypovolemic shock, sepsis, and other severe conditions. A normal range is 0.5 to 0.7. ^33^ It has been shown to be predictive of mortality in trauma patients (those with a value above 0.9 having higher mortality rates^34^ ).

## Methods 

**Data source**

Data for this study was pulled from the trauma registry of UT Health East Texas (UTHET) – Tyler, Texas. UTHET is the only Level I trauma center in the Texas Public Health Region 4/5N. As such the trauma registry there is the most complete registry in the region. Data was pulled for the time frame of January 2011 - October 2016. This paper examined all injuries which occurred as a result of ATV injuries in the region, Northeast Texas. A total of 2,204 original entries were in the original trauma registry data files. Only patients who were alive upon arrival were included in the data. 

**Data treatment**

HIPAA information was stripped from the raw data files. Patients that were determined to be duplicate entries were deleted. In addition, considering a duplication of a patient entry into the trauma registry, only the last admission and discharge of each patient was retained for final data analysis. If a smaller emergency facility triaged the initial patient prior to transfer to a larger facility (i.e., UTHET), only the last data was retained for final data analysis. Only entries from ATV-related injuries were retained. This was determined by review of E-codes, V-codes, and mechanism of injury entries. The E-codes considered for this analysis were 816.0, 819.1, 821.0-9, 823.0, 825.0-1, and 849.1 while the V-codes included 86. (09, 1, 19, 39, 51, 59 and 69). The final count of all eligible trauma registry entries retained was 543. Data was then cleaned and coded for analysis.

**Outcome and other variables**

The outcome measures of interest were hospital length of stay (LOS) captured in days and hospital charges captured in dollar amounts.

The patient demographics captured by UTHET trauma registry and included in this analysis were age (in years), sex and race.

General injury characteristics included the environmental site (location) where the injury occurred, the injured body part, the injury severity and the use of protective devices. The environmental sites of injury were grouped into a categorical variable (injury location) with recreation or sport site, home and farm/street/highway/other as different categories. Some patients had injuries on multiple body parts. In defining the injured body part variable, primary consideration was given to body parts with higher risk of severe injury or fatality. The primary injury was originally reported as head/face/neck, trunk/thorax/pelvis, trunk/extremities and extremities injuries. Since it was difficult to delineate trunk from thorax/pelvis and from extremity, these were all grouped into one category such that a binary categorical variable which constituted of head/face as one category and trunk/thorax/pelvis/extremity as another defined the injured body part variable. Injury severity was captured in terms of the ISS and GCS recorded as scale (continuous variables). The use of various protective devices by these patients prior to injury were noted. These were grouped in a categorical variable with the following levels: helmet, padding, safety belt/harness and none.

Hospital/ED-related factors included the facility where the patient was treated, the means by which the patient was transported to this facility, the initial ED vital signs, the means of payment for treatment and patient condition upon discharge. Some patients were seen/treated at multiple facilities, but as noted earlier, only information on the last facility was retained and for the purpose of this analysis, these were grouped into UTHET and other. UTHET was segregated and of particular interest because it is the only region 1 trauma center in East Texas. The means of transportation to the treatment facility was captured as emergency medical services (EMS) or other. The initial ED vital signs included respiration rate (ED RR), heart rate (ED Pulse), and systolic blood pressure (ED SBP). Shock index was also an important variable of interest related to ED. This was computed as the ratio of ED pulse and ED SBP. The means of payment for treatment was also recorded as private insurance, public insurance, self-pay and other. Patient condition upon discharge was noted as alive expecting full recovery, alive expecting moderate recovery, alive expecting severe disability, transferred to another acute care facility or dead.

All the categorical variables described above were further dummy-coded for analytical purposes.

**Statistical analysis**

Data analysis was performed using the IBM SPSS Statistics for Windows, Version 25.0. (IBM Corp, Armonk, NY, USA).

Initial exploratory analyses of the distribution of the outcome measures of interest, hospital LOS and charges, revealed that while LOS followed a normal distribution, charges did not. As such, the natural logarithm transformation (allowing for easy interpretation of regression coefficients) was applied on charges to make the distribution normal. Separate multiple linear regression models were fitted for each of these outcomes with all potential predictors including patient demographics, general injury characteristics and hospital/ED- related factors, using the stepwise approach. Statistical significance was assessed at the 5% level of significance. 

The general descriptive statistics (means/standard deviation for all continuous variables and frequency distribution for all categorical variables) are depicted on Table 1. The sample was predominantly male (68.3%) and white (85.3%), with the mean age of all participants 29.63 ± 16.5 years. A plurality of the injuries was due to sport/recreation (45.8%), affecting mostly the trunk/thorax/pelvis/extremities (64%). Over 28% were minor injuries with a mean ISS of 9.01 ± 7.5 and GCS of 14.32, the use of no protective devices was reported by most (67.5%) of the patients.

**Table 1:** General descriptive statistics of the patients with all-terrain vehicle-related injuries in rural Northeast Texas.

	n (%)
**Patient demographic characteristics**	
Age in years [Mean(SD)]*	29.63(16.5)
**Sex**	
Female	173(31.7)
Male	372(68.3)
**Race**	
White	465(85.3)
Black	47(8.6)
Hispanic	17(3.1)
Other	14(2.6)
**Injury characteristics Location of injury**	
Recreation/sport site	727(45.8)
Home	259(6.3)
Farm /street/highway	600(37.8)
**Injured body part**	
Head/Face/Neck	196(36.1)
Trunk/thorax/pelvis/extremities	321(58.9)
Missing	27(5.0)
**Tools to Measure Trauma Severity [Mean(SD)]**	
ISS	9.01 (7.5)
GCS	14.32 (2.5)
**Use of Protective devices**	
Helmet	20(3.7)
Padding	1(0.2)
Safety Belt/Harness	7(1.3)
None	368(67.5)
Missing	149(27.3)
**Hospital/ED-related factors Facility**	
UTHET	134(24.6)
Other	411(75.4)
**Transportation**	
Emergency medical services	242(44.4)
Private vehicle	35(6.4)
Other	268(49.2)
**Initial ED vital signs [Mean(SD)]**	
ED respiration rate	18.78(5.8)
ED pulse	91.20(20.7)
ED systolic blood pressure	130.05(23.8)
Shock index	0.72(0.3)
**Means of payment **	
Private Insurance	264(48.4)
Public Insurance	80(14.7)
Self-Pay	141(25.9)
Other	60(11)
**Condition at Discharge**	
Alive, Full Recovery	373(68.4)
Transferred to another acute care	144(26.4)
Alive, moderate recovery	13(2.4)
Alive, expected severe disability	3(0.6)
Dead	8(1.5)
**Outcome of interest [Mean (SD)] **	
LOS (days)	3.78(5.7)
Ln Charges ($)	10.14(1.2)

*SD=standard deviation; ED=Emergency department, ISS= injury severity score; GCS=Glasgow coma scale; UTHET= UT Health East Texas; Missing Use of Protective Devices Info on 149 individuals.

Only 24.6% of the patients were treated at UTHET with 44.4% using the EMS. The mean shock index was 0.72 ± 0.3. All the initial ED vital signs are shown onTable 1. The plurality (48.4%) of patients had private/commercial insurance, with 25.9% being self-pay. Upon discharge most (68.4%) were alive, expecting full recovery with only a very small proportion expecting severe disability (0.6%) or dead (1.5%). A substantial proportion (26.4%) was transferred to another acute care facility. The mean LOS and hospital charges were 3.78 ± 5.7 days and $45,582 ± $10,167.

## Results

Table 2 summarizes the final regression models for LOS and hospital charges. LOS was predicted positively by ISS, ED RR and facility at which patient were treated and negatively by GCS with only about 26% of the total variation in LOS explained by these variables (adjusted R^2^=0.259). According to Table 2, the average LOS increased by 0.302 days for every unit increase in ISS and decreased by 0.736 days for every unit increase in GCS. Patients treated at the level 1 trauma center (UTHET) had over 2 days longer LOS compared to those treated somewhere else. Each unit increase of initial ED RR corresponded with an average increase of LOS by 0.202 day. 

**Table 2:** Coefficient estimates and standard errors (SE) of potential predictors of hospital length of stay and charges of patients with all-terrain vehicle- related injuries in rural Northeast Texas.

	Coefficient estimate (SE)*
**Predictors of hospital length of stay**	
ISS	0.302(0.048)
GCS	-0.736(0.165)
UTHET	2.103(0.746)
ED Respiration rate	0.202(0.101)
**Predictors of natural log of hospital charges**	
Age	0.012(0.004)
ISS	0.030(0.008)
GCS	-0.064(0.025)
UT Health -Tyler	0.408(0.142)
Emergency medical services transport	0.598(0.208)
ED pulse	0.015(0.003)

ISS= injury severity score; GCS=Glasgow comma scale, UTHET=University of Texas Health East Texas; ED=emergency department, Hospital length of stay Adjusted R^2 = 0.259; Natural log of hospital charges Adjusted R^2 = 0.263. *: Only factors with statistically significant (p <0.05) coefficient estimates are presented.

Hospital charges were predicted positively by age, ISS, facility of treatment, means of transportation, and ED pulse and negatively by GCS, with over 2% of the variation in the natural logarithm of charges explained by these variables (adjusted R^2^ = 0.263). Each additional year in age was associated with a 1% increase in charges. Also, each unit increase in ISS was associated with an average increase of approximately 4.5% in hospital charges while a unit increase in GCS resulted in a decrease of 6.3% in charges. Cases at UTHET incurred on average almost 50% higher charges than the other hospitals (exp0.408 = 50.38%). Likewise, those transported by EMS incurred 82.75% higher charges than those who did not arrive by ambulance. For each unit increase in pulse rate, there was an associated approximately 1% increase in charges. These were all statistically significant at p <0.05.

## Discussion

Several findings were contradictory to what was previously hypothesized. It was interesting to note that several variables expected to be statistically significant were not. Specifically, it was expected that the use of protective devices would predict hospital length of stay and charges. However, this relationship was not observed. That could be a result of the significant number of patients who either didn’t wear protective devices or it was not reported to the trauma registry. There may not have been enough cases to generate statistical significance. Although other studies have found that wearing protective equipment, especially helmets,^6^ is correlated with a reduction in serious injuries, this data does not indicate that it has any bearing on hospital LOS or costs. Complementary to the literature, the majority of patients analyzed in this study were adults, contrasting the literature’s focus primarily on pediatric populations. Although it is understandable why there has been such a focus on children in the past, adult ATV injuries warrant further evaluation due to the significant number of cases the authors were able to find.

Nonetheless, some of the results were expected. The facility of treatment (being a Level 1 trauma center) was a predictor of both length of stay and charges. This might have been due to the fact that more serious cases ended up at the Level 1 trauma center (UTHET). Likewise, those with higher ISS and lower GCS scores are typically more serious cases and therefore, probably require greater interventions. It was interesting to note that respiration was predictive only for length of stay and heart rate and age were only predictive for charges. The age variable may be due in part to the more severe impact that traumatic injuries can have on older adults.^35^ The fact that the mode of payment was not a statistically significant predictor of length of stay means that the physicians were treating the patients without regards to payment ability, which is what should be expected. 

There are some limitations to this study. As is common with most hospital data which was not initially collected for research purposes it is possible that data was not recorded consistently or that errors were introduced by the various staff recording the data differently. There was a switch from ICD-9 to ICD-10 in the middle of the study duration and this limits the ability of the investigators to compare injuries directly over the course of the entire study. 

## Conclusion

The authors hope that this study can contribute to the creation of better models to predict a wide range of outcomes, not just length of stay or charges, in ATV injury patients. The current study found that injury severity (measured in terms of the Injury Severity Score and the Glasgow Coma Scale) and the facility where the patient was treated (being a Level 1 trauma center) were predictive for both hospital length of stay and hospital charges. It also found that the initial emergency department vital signs were useful in predicting length of stay (respiration rate) and hospital charges (heart rate). The results showed that commonly collected variables upon arrival to the ED can be useful in predicting LOS and hospital charges. This study helps to add to the literature regarding ATV related injuries in both adults and children and the healthcare costs associated with such injuries. Our average cost incurred was much higher ($45,582) than some of the previous studies have found ($8,802.90.),^23^ which indicates that further research should be done to explore why there was such as variation in costs. We hope that this sparks a conversation on the importance of examining adult ATV injuries in the research literature. 
